# Supplementary material for: Heavy tails and pruning in programmable photonic circuits for universal unitaries
Source: Nat Commun. 2023 Apr 3;14:1853. doi: 10.1038/s41467-023-37611-9 (PMC10070444; doi:10.1038/s41467-023-37611-9)
Supplement: Supplementary file 1 — Supplementary Information [file 41467_2023_37611_MOESM1_ESM.pdf]

Supplementary Information for "Heavy tails and pruning in programmable photonic circuits for universal unitaries"

Sunkyu Yu[1†] and Namkyoo Park[2*]

[1]Intelligent Wave Systems Laboratory, Department of Electrical and Computer Engineering, Seoul National University, Seoul 08826, Korea

[2]Photonic Systems Laboratory, Department of Electrical and Computer Engineering, Seoul National University, Seoul 08826, Korea

E-mail address for correspondence: [†]sunkyu.yu@snu.ac.kr, [*]nkpark@snu.ac.kr

Note S1. Differentiated transformations of off-diagonals through nulling processes

Note S2. Uniform distribution of φ-rotations

Note S3. Heavy-tailed distribution: Power-law model with an exponential cutoff

Note S4. Heavy-tailed distribution: Log-normal model

Note S5. Non-heavy-tailed distribution: Exponential model

Note S6. Universal architecture for n = 64

Note S1. Differentiated transformations of off-diagonals through nulling processes

Figure S1 describes the first 4 steps of nulling the off-diagonal elements in the 5-degree random unitary matrix U_5. Each nulling process is achieved with T_m^l, which has the SU(2) block matrix for the mth and (m+1)th channels (green squares in T_m^l or $(T_m^l)^\dagger$). The nulling processes are composed of two forms of nulling to preserve the nulled elements (black squares in U and U') from the prior steps: $U = U'(T_m^l)^\dagger$ (orange arrows in Figs S1a and S1d) and $U = T_m^l U'$ (blue arrows in Figs S1b and S1c), where U and U' are the transformed unitary matrix at the current and previous steps, respectively. Each form sets one of the off-diagonal elements to be zero: (l, m) matrix element with $U'(T_m^l)^\dagger$ and (m+1, l) matrix element with $T_m^l U'$.

During the nulling of the target off-diagonal element with the designed θ and φ, the other elements at the nearby rows or columns (orange or blue boxes in U and U') inevitably undergo SU(2) operations. Because the values of the previously nulled off-diagonal elements are maintained, each matrix element eventually undergoes a different number of SU(2) transformations; elements that are nulled earlier get fewer transformations. Such a discrepancy in the numbers of SU(2) transformations applied to the off-diagonal elements is general for any design strategies that adopt a series of nulling processes for the factorization of the target unitary operation, such as the Reck design[1]. However, the specific number of SU(2) transformations of each matrix element can differ depending on the nulling algorithm.

As described in the Clements design[2], nulling processes are applied only to the lower triangular off-diagonal elements because a unitary triangular matrix is diagonal. After the entire nulling process, we employ the relationship $(T_m^l)^\dagger D = D' T_m^l$, where D is the diagonal matrix resulting from the nulling processes, and D' is its transformation with T_m^l. This relation that is valid for Hermitian systems is used to derive Eq. (1) in the main text.

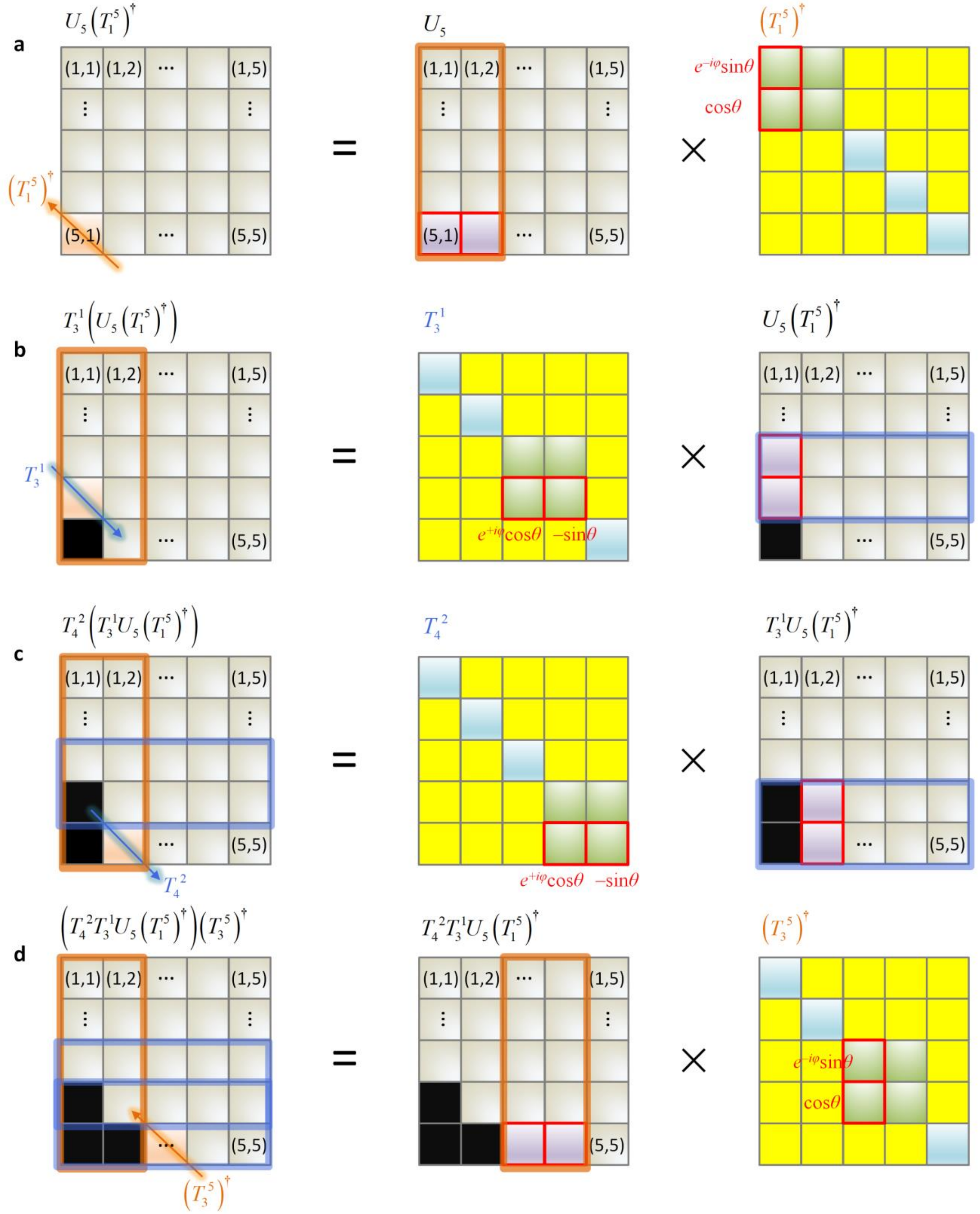

Fig. S1. Why are nulling processes unequal? a-d, The first 4 transformations of the 5-degree random unitary matrix U_5: $U = U_5(T_1{}^5)^\dagger$ (**a**), $U = T_3{}^1U' = T_3{}^1U_5(T_1{}^5)^\dagger$ (**b**), $U = T_4{}^2U' = T_4{}^2T_3{}^1U_5(T_1{}^5)^\dagger$ (**c**) and $U = U'(T_3{}^5)^\dagger = T_4{}^2T_3{}^1U_5(T_1{}^5)^\dagger(T_3{}^5)^\dagger$ (**d**). U and U' are the transformed unitary matrices at

the current and previous steps of the nulling processes. Orange arrows in **a** and **d** and blue arrows in **b** and **c** represent the nulling of the target off-diagonal element in U. Purple and green squares surrounded by red boxes represent the multiplied elements in U' and $T_m{}^l$ (or $(T_m{}^l)^\dagger$), respectively, to obtain the target element in U. This multiplication for nulling defines the values of θ and φ. Black squares in U and U' denote nulled elements. The orange and blue boxes in U and U' are matrix elements that undergo SU(2) transformations from $T_m{}^l$. Yellow and blue squares in T_{ml} or $(T_m{}^l)^\dagger$ represent values of 0 and 1, respectively.

Note S2. Uniform distribution of φ-rotations

Figure S2 shows an example of the φ distribution for U_{128}, which is obtained from the uniform sampling of the U(128) group with the Haar measure[3]. The ensemble includes 8128 values of φ, which is half the number of off-diagonal elements. As shown in the linearized CCDF plot on the linear scale (Fig. S2a) and the almost flat distribution of the PDF (Fig. S2b), φ possesses a uniform distribution in sharp contrast to the heavy-tailed distribution of θ. This result proves that the differentiated behaviours of θ and φ on the Bloch sphere (Figs 2b and 2c in the main text) result in the apparent distinction in their statistics.

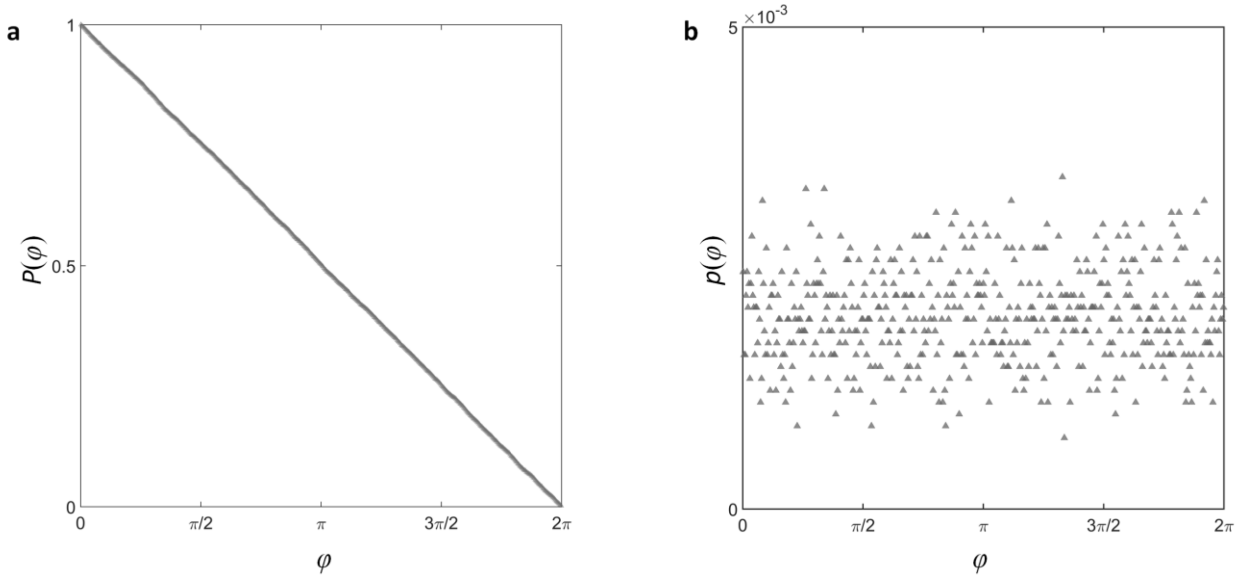

Fig. S2. Statistical distributions of φ-rotations. a,b, Distributions of φ described by its CCDF (**a**) and PDF (**b**). The range of φ is $\varphi \in [0, 2\pi)$. All the scales of the axes are linear.

Note S3. Heavy-tailed distribution: Power-law model with an exponential cutoff

Although the power-law model is one of the well-established heavy-tailed models[4-6], many empirical data sets in natural, technological, or social systems are not perfectly described by the ideal power-law model. To handle imperfect types of power-law-like data sets, some truncated forms of the power-law model, such as the power-law model having a cutoff, can be successful.

We apply the power-law model with an exponential cutoff to the θ-distributions of programmable photonic circuits with different degrees n. The model parameters $\{q_s\} = \{\alpha_c, \lambda_c\}$ and the lower-bound parameter $\theta_{c,\min}$ are obtained from the minimization of $-L$ and the Kolmogorov–Smirnov test, as described in the Methods section. Similar to the results for the power-law model (Figs 2f and 2g in the main text), the model parameters and the lower-bound parameter become consistent with the increase in n, which shows the validity of the model (Fig. S3). Notably, the model parameters thoroughly describe the crossover in our problem. The increase in the power-law exponent α_c and the decrease in the cutoff exponent λ_c (Figs S3a and S3b) demonstrate that the tails of the distribution become heavier with a larger n, which is also confirmed by the increasing range of model validity (Fig. S3c).

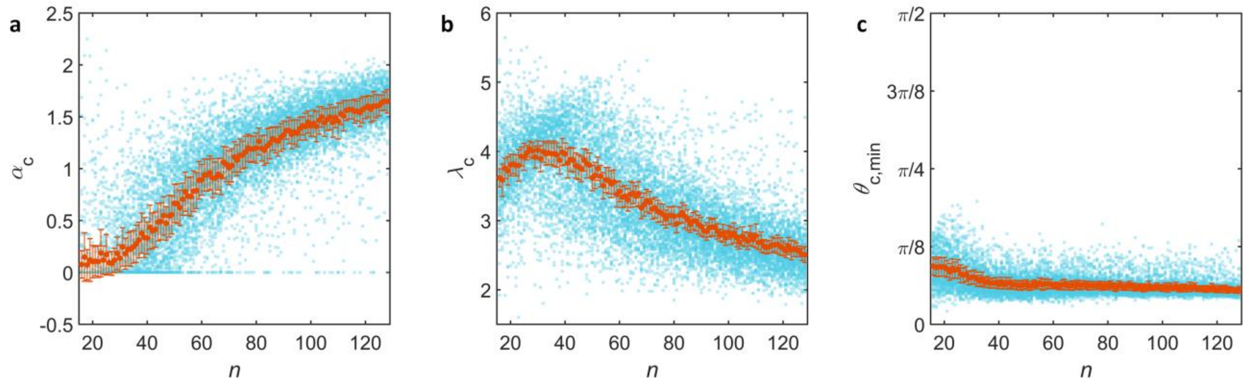

Fig. S3. Model parameters of the power-law distribution with an exponential cutoff. a-c, The variations of the model estimators for different n: α_c (**a**), λ_c (**b**) and $\theta_{c,\min}$ (**c**). Each blue point represents a realization, and orange markers and error bars show the average and root-mean-square error (RMSE) of 100 realizations at each value of n, respectively.

Note S4. Heavy-tailed distribution: Log-normal model

We also study another form of the heavy-tailed model: the log-normal distribution, which can originate from the product of multiple independent positive random numbers[4]. Figure S4 shows the n-dependent model parameters $\{q_s\} = \{\mu, \sigma\}$ obtained from the minimization of $-L$ (Methods section), which demonstrates the model validity for large-scale photonic circuits with the enhanced consistency of μ and σ.

As a crossover distribution between a power-law model and an exponential model, the log-normal distribution with large σ resembles a power law due to the exponential growth of $<\theta^2>$, where <…> is the ensemble average[4]. In this context, Fig. S4b further demonstrates a more power-law-like (or heavy-tailed) nature of the θ-distribution in larger-scale photonic circuits.

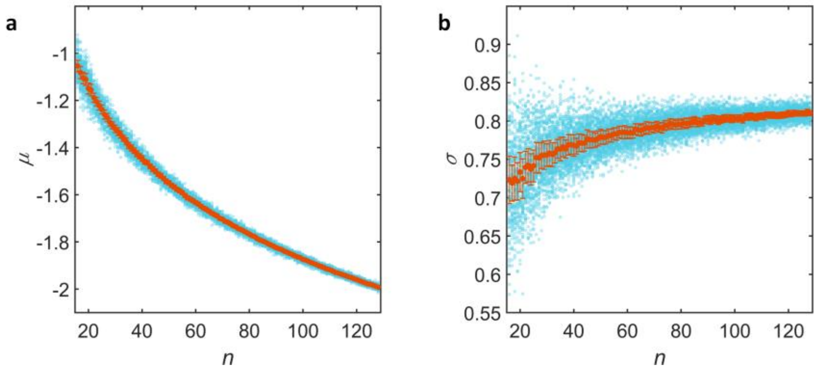

Fig. S4. Model parameters of the log-normal distribution. a,b, The variations of the model estimators for different n: μ (**a**) and σ (**b**). Each blue point represents a realization, and orange markers and error bars show the average and root-mean-square error (RMSE) of 100 realizations at each n, respectively.

Note S5. Non-heavy-tailed distribution: Exponential model

While we demonstrate the validity of multiple heavy-tailed models—power-law, power-law with an exponential cutoff and log-normal models—for θ-distributions, it is instructive to compare the use of a distribution other than a heavy-tailed model, such as the exponential distribution, to fit the data[4,7]. Figure S5 shows the model parameter $\{q_s\} = \{\lambda_e\}$ and the lower-bound parameter $\theta_{e,min}$ obtained from the MLE relation and the Kolmogorov–Smirnov test (Methods section). As shown in Fig. S5a, the model is inconsistent with the significant deviations from the average of λ_e. While the level of such errors from the model is maintained regardless of n values, the lower bound $\theta_{e,min}$ increases with n (Fig. S5b), demonstrating that the exponential model does not provide a valid fit to θ-distributions.

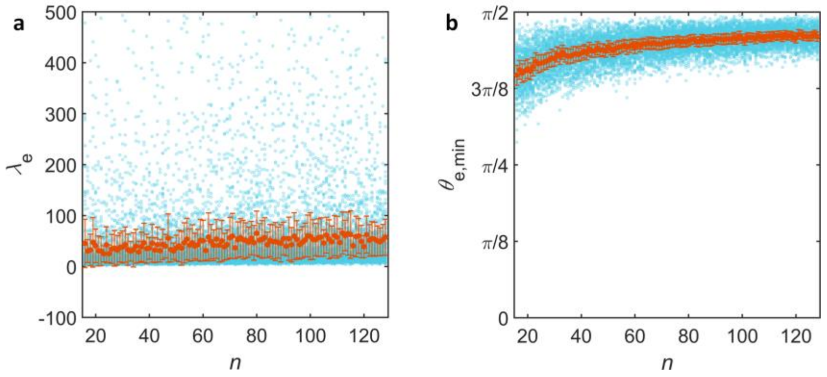

Fig. S5. Model parameters of the exponential distribution. a,b, The variations of the model estimators for different n: λ_e (**a**) and $\theta_{e,min}$ (**b**). Each blue point represents a realization, and orange markers and error bars show the average and root-mean-square error (RMSE) of 100 realizations at each n, respectively.

Note S6. Universal architecture for *n* = 64

Figures S6a and S6b describe the universal architectures defined by <$\theta_{m,l}$> and <$\varphi_{m,l}$>, respectively, for n = 64. Similar to the results of n = 16 and n = 32 (Figs 4a and 4b in the main text), we again confirm the inhomogeneous distribution of <$\theta_{m,l}$> in contrast to the uniform distribution of <$\varphi_{m,l}$>.

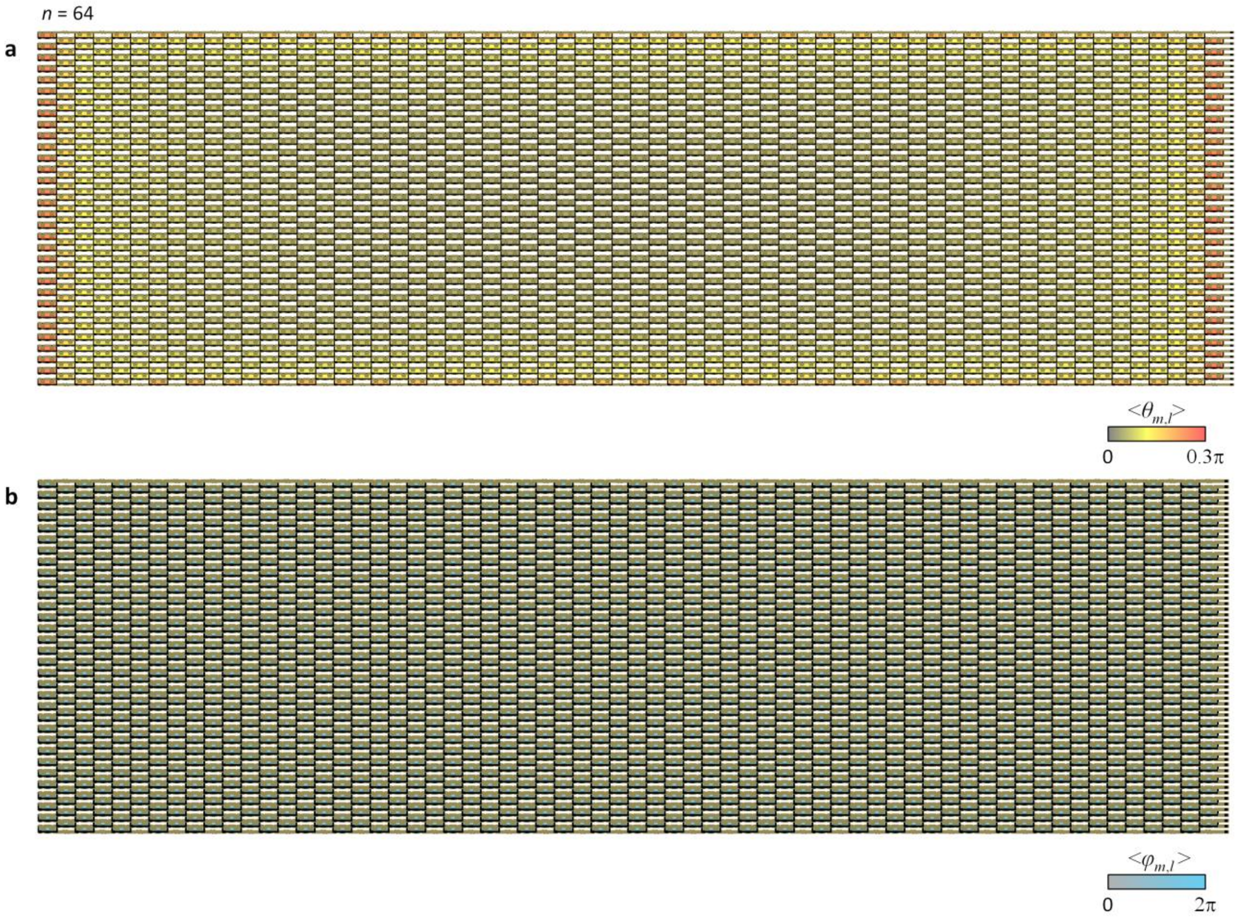

Fig. S6. Universal architecture of programmable photonic circuits for U_{64}. a,b, The averages <$\theta_{m,l}$> (**a**) and <$\varphi_{m,l}$> (**b**) for the photonic circuits of 100 U_n realizations with n = 64. We set the upper bound of the colormap in **a** to be 0.3π for better visibility.

Supplementary References

1. Reck, M., Zeilinger, A., Bernstein, H. J. & Bertani, P. Experimental realization of any discrete unitary operator. *Phys. Rev. Lett.* **73**, 58 (1994).
2. Clements, W. R., Humphreys, P. C., Metcalf, B. J., Kolthammer, W. S. & Walmsley, I. A. Optimal design for universal multiport interferometers. *Optica* **3**, 1460-1465 (2016).
3. Haar, A. Der Massbegriff in der Theorie der kontinuierlichen Gruppen. *Ann. Math.* **34**, 147-169 (1933).
4. Barabási, A.-L. *Network science* (Cambridge university press, 2016).
5. Barabási, A.-L. & Bonabeau, E. Scale-Free Networks. *Sci. Am.* **288**, 60-69 (2003).
6. Voitalov, I., van der Hoorn, P., van der Hofstad, R. & Krioukov, D. Scale-free networks well done. *Phys. Rev. Res.* **1**, 033034 (2019).
7. Clauset, A., Shalizi, C. R. & Newman, M. E. Power-law distributions in empirical data. *SIAM Rev.* **51**, 661-703 (2009).
