# Supplementary material for: Heavy tails and pruning in programmable photonic circuits for universal unitaries
Source: Nat Commun. 2023 Apr 3;14:1853. doi: 10.1038/s41467-023-37611-9 (PMC10070444; doi:10.1038/s41467-023-37611-9)
Supplement: Supplementary file 3 — Description of Additional Supplementary Files [file 41467_2023_37611_MOESM3_ESM.pdf]

Description of Additional Supplementary Files

Supplementary Code 1: This code file includes the Supplementary codes for generating the data of Figs. 2–5.
